# Nine New Species of *Aleiodes* Wesmael Reared at Yanayacu Biological Station (Hymenoptera: Braconidae: Rogadinae) in Eastern Ecuador

**DOI:** 10.1673/031.009.3701

**Published:** 2009-06-02

**Authors:** Andrew C. Townsend, Scott R. Shaw

**Affiliations:** ^1^ Yanayacu Biological Station, Napo prov., Cosanga, Ecuador; ^2^ Department of Renewable Resources, University of Wyoming

## Abstract

Nine new species of *Aleiodes* (Braconidae: Rogadinae) are described and illustrated: *A. aclydis, A. albiterminus, A. arbitrium, A. atripileatus, A. capillosus, A. greeneiyi, A. nebulosus, A. speciosus* and *A. stilpnos.* Because of the difficulties in distinguishing Neotropical species that belong to the *circumscriptus* and *gastritor* species-groups, a larger species-group combining the two, termed the *circumscriptus/gastritor* species-group, is created. The new species described in this study belonged to the *seriatus, albitibia, gressitti,* and *circumscnptus/gastritor* species-groups, respectively. *Aldodes capillosus* represents the first Neotropical species belonging to the gressitti species-group. Of the 34 previously described Neotropical species in *Aldodes,* only 13 have known biologies. The *Aleiodes* species in this study were reared from the families Geometridae and Noctuidae, two of the most common host families of other *Aleiodes* species worldwide.

## Introduction

*Aleiodes* Wesmael, a species rich genus of the rogadine braconids, exhibits a cosmopolitan distribution but is particularly prevalent in the New World (Shaw et al, 1997; Shaw, 1997; Delfin-Gonzalez and Wharton, 2002). This pattern of species richness was previously overlooked until recent authors recognized that many species previously classified as *Rogas* Nees should be transferred to *Aleiodes* (van Achterberg, 1982, 1985, 1991; Marsh, 1989; M. Shaw and Huddleston, 1991; Shaw, 1993, 1995; Shaw et al., 1997). Worldwide, approximately 225 *Aleiodes* species are known (Delfin-Gonzalez and Wharton, 2002), with 90 of those being found in the Nearctic. In contrast, only 34 have been described from the Neotropics. Of those 34, only 13 have known biologies (van Achterberg and Penteado-Dias, 1995; Shaw et al., 1997; Marsh and Shaw, 1998; Marsh and Shaw, 1999; Fortier, 2000; Delfin-Gonzalez and Wharton, 2002; Shaw et al. 2006). The difference in *Aleiodes* known diversity between Nearctic and Neotropical regions is likely due to the fact that Nearctic species have been well studied, whereas little is known about the Neotropical fauna (Shaw, 1997; Shaw et al., 1997; Shaw et al., 1998a; Marsh and Shaw, 1998; Shaw et al., 1998b; Marsh and Shaw, 1999; Marsh and Shaw, 2001; Marsh and Shaw, 2003; Shaw et al., 2006; Delfin-Gonzalez and Wharton, 2002). *Aleiodes* are koinobiont endoparasitoids of Lepidoptera larvae; thus the host continues to develop after parasitism (M. Shaw and Huddleston, 1991). *Aleiodes* species primarily attack relatively large, exposed-feeding caterpillars. They have been reared from the following families: Arctiidae, Bombycidae, Choreutidae, Drepanidae, Gelechiidae, Geometridae, Hesperiidae, Incurvariidae, Lasiocampidae, Limacodidae, Lycaenidae, Lymantridae, Lyonetiidae, Noctuidae, Notodontidae, Nymphalidae, Psychidae, Pyralidae, Sphingidae, and Tortricidae (M. Shaw and Huddleston, 1991; Shaw, 1995, 1997; Fortier, 2000; Delfin-Gonzalez and Wharton, 2002). Most species attack second or third instar caterpillars as adults and the larvae emerge from and kill the host in later instars. *Aleiodes* is unique in that pupation occurs within the shrunken, hardened remains of the host caterpillar, which often is referred to as a “mummy”. The host mummy is glued to the host plant or some other substrate via a fluid that is exuded by the wasp larvae through the antero-ventral portion of the host thorax. Sometimes the amount of fluid exuded is insufficient to effectively attach the mummy to a substrate. Upon emergence from the mummy, the parasitoid cuts a circular, postero-dorsal hole. Although the host mummy is lined with parasitoid silk, structural support for the mummy is apparently provided by the premature formation of host pupal cuticle, presumably caused in response to *Aleiodes* larvae (M. Shaw and Huddleston, 1991; Shaw et al., 1997; Quicke and M. Shaw, 2005). The form of the mummy caused by a particular *Aleiodes* species in its host is characteristic and can be used to separate different wasp species (Shaw et al., 1997; Shaw, 2006). The biology of most described species in the Nearctic and Neotropical regions is completely unknown (van Achterberg and Penteado-Dias, 1995; Shaw, 1997; Shaw et al., 1997; Shaw et al., 1998a; Shaw et al., 1998b; Marsh and Shaw, 1998; Marsh and Shaw, 1999; Fortier, 2000; Marsh and Shaw, 2001; Marsh and Shaw, 2003; Shaw et al., 2006).

In their revision of Nearctic *Aleiodes,* S. Shaw, P. Marsh and J. Fortier have published a series of papers broken up by species-groups (Shaw et al., 1997; Shaw et al., 1998a; Marsh and Shaw, 1998; Shaw et al., 1998b; Marsh and Shaw, 1999; Marsh and Shaw, 2001; Marsh and Shaw, 2003; Shaw et al., 2006) based on a phylogenetic analysis (Fortier and Shaw, 1999). In the earliest of those, Shaw et al. (1997) provided a key to species-groups that, although written for Nearctic taxa, applies for most Neotropical species as well. Nevertheless, members of the *circumscriptus* and *gastritor* species-groups reared in this study do not exhibit some diagnostic features described by Fortier and Shaw (1999) and Shaw et al. (1997). The *gastritor* species-group is defined by having a pronotal medial length of less than or equal to 30% of head length (Fortier and Shaw, 1999). Yet members of the *circumscriptus* species-group, including *A. autographae* (Viereck) and *A. circumscriptus* (Nees) can also have short pronotal lengths. The *circumscriptus* species-group is defined by having metasomal tergite 2 yellow medially and black laterally (Fortier and Shaw, 1999). However, color is a variable trait within Braconidae and may not be phylogenetically conserved across all members of the *circumsmptus* species-group. In addition, the *circumsmptus* and *gastritor* species-groups can be distinguished by the size of their ocelli. Members of the *circumsmptus* speciesgroup have small ocelli and the ocell-ocular distance is larger than the width of the lateral ocelli. By contrast, members of the *gastritor* species-group have larger ocelli and the ocell-ocular distance is smaller than the width of the lateral ocelli (Shaw et al., 1997). However, ocellus size is often associated with a species being either nocturnal or diurnal, so selection with respect to activity period could produce ocelli diverging in size. Some species can be separated into their respective group definitively, while others are of uncertain placement because of variation in these characters. We recommend that Neotropical species of these two groups be lumped into one larger group, here termed the *circumscriptus/gastritor* species-group. Hopefully, in the future we may find new characters, or molecular markers, that will allow a more definitive assignment of these species.

Although the Nearctic *Aleiodes* are currently being revised (Shaw et al., 1997; Shaw et al., 1998a; Marsh and Shaw, 1998; Shaw et al., 1998b; Marsh and Shaw, 1999; Marsh and Shaw, 2001; Marsh and Shaw, 2003; Shaw et al., 2006), relatively little work is being done on Neotropical species (van Achterberg and Penteado-Dias, 1995; Delfin-Gonzalez and Wharton, 2000; Delfin-Gonzalez and Wharton, 2002). A notable exception is the work of Angelica Penteado-Dias, who is currently studying *Aleiodes* of the coastal forests of Brazil. However, much work remains to be done; it has been estimated that there are over 200 undescribed species *of Aleiodes* in the Neotropics (Delfin-Gonzalez and Wharton, 2002). Were all to be described, this would nearly double *Aleiodes* in size.

## Materials and Methods

Specimens for this study were reared as part of the *Caterpillars and Parasitoids of the Eastern Andes of Ecuador* project (Dyer et al, 2008) at the Yanayacu Biological Station, Napo province (Greeney, 2008). Caterpillars were sampled by walking through various habitats inspecting herbs, shrubs and trees up to a height of approximately 2.5 m. Caterpillars were collected in clear plastic bags with their food plant, assigned an identification code and transported to the rearing shed at the biological station. Caterpillars and host plants were identified and recorded. Rearing took place in plastic bags in an open-air, covered shelter in which the caterpillars were exposed to ambient temperatures and day length. Frass and unused or decaying plant material were removed every other day. New plant material was provided as necessary. While cleaning out the bags, the caterpillars were inspected to note the date of caterpillar pupation or date of parasitoid pupation. Parasitoid pupae were inspected daily for emergence. All emerging adult parasitoids were kept with the original code given to the caterpillar to preserve host and host plant data. The parasitoids were preserved in alcohol and transferred to the University of Wyoming where they were dried and point mounted for identification.

**Figure 1.**
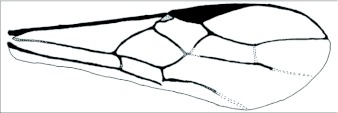
General fore wing diagram modified from Sharkey and Wharton (1997).

**Figure 2.**
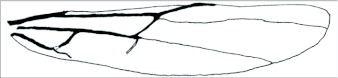
General hind wing diagram modified from Sharkey and Wharton (1997).

The definition *of Aleiodes* used in this paper follows that of van Achterberg (1991), Shaw (1993), Shaw et al. (1997) and Fortier and Shaw (1999). Specimens were identified to species-group using the key to *Aleiodes* species-group in Shaw et al. (1997). Because of the difficulties in distinguishing between the *circumscriptus* and *gastritor* speciesgroups, species from either of these were placed in a single *circumscnptus/gastritor* species-group.

Terminology follows that used for *Aleiodes* by Shaw et al. (1997). Microsculpture terminology mostly follows Harris (1979). Wing veins are named in accordance with Sharkey and Wharton (1997). Generalized wing diagrams have been included here for clarity (Figures 1 and 2).

## Key to the Species of *Aleiodes *Reared from the Northeast Andes of Ecuador (see p. 4)

**Figure 3.**
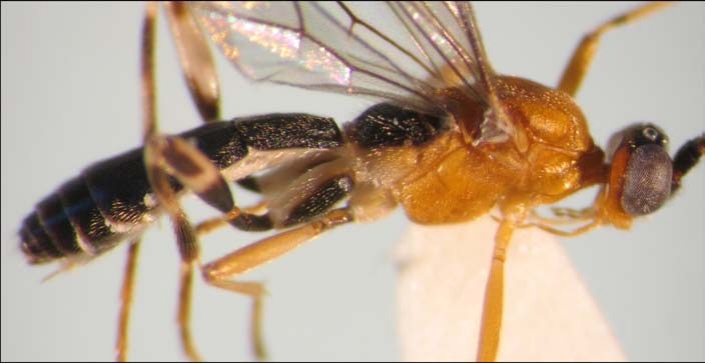
Lateral habitus of A *aclydis* n sp. Length, 6.1 mm.

**Figure 4.**
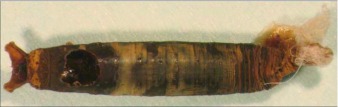
Host mummy of *A. aclydis* n sp. Length, 11.0 mm.

***Aleiodes aclydis* Townsend, new species (Figures 3, 4)**
♀. ***Body color***: face, frons, ocellar triangle and anterior portion of vertex black, remainder of head honey brown; mandible mostly honey brown, tip dark brown; maxillary palpus and labial palpus honey brown; antenna silverblack; fore leg uniformly light brown; coxa of mid leg off white; trochanter and trochantellus of mid leg off white to light brown dorsally, dark brown to black ventrally; femur, tibia and tarsus of mid leg off white to light brown; coxa of hind leg off white basally, black apically; trochanter and trochantellus of hind leg mostly black, apical tip of trochantellus off white; ground color of hind femur off white with irregular black markings basally and at apex, black markings more extensive on outer margin; ground color of tibia dark brown to black with off white markings basally and medially; tarsus of hind leg light brown; pronotum, mesonotum, mesopleuron and scutellum honey brown; propodeum black; metasomal terga black; ovipositor sheath black, ovipositor light brown (Figure 3).
***Body length***: = 6.1 mm; fore wing length = 6.2 mm.
***Head:*** antenna comprising 42 antennomeres, all flagellomeres roughly equal to or slightly longer than 2.0 × width; malar space short, length about 0.7 × width of mandibular base; occipital carina weak or absent at vertex, well defined elsewhere, except at extreme base where it is weak, but still meeting hypostomal carina; oral space small and circular, diameter equal to malar space; clypeus swollen; ocellus large, ocell-ocular distance 0.4 × diameter of lateral ocellus; head granulate; maxillary palpus not swollen; tips of mandibles touching when closed.
***Mesosoma***: pronotum granulate; mesonotum granulate, notauli with irregular fovea anteriorly, incomplete posteriorly; scutellum mostly granulate with irregular fovea postero-laterally; mesopleuron granulate, sternaulus present, but short, epicnemial carina weak; propodeum granulate-rugose, median carina absent.
***Legs***: tarsal claw simple.
***Wing***:(*fore* wing) vein r about 0.33 × vein 3RSa; vein RS+Mb about equal in length to vein r; vein 1cu-a beyond vein 1M by length about 3.0 × vein 1cu-a. (Hind wing) vein RS slightly sinuate, thus marginal cell narrowest in middle; vein r-m about 0.9 × vein 1M; vein M+CU longer than vein 1M; vein m-cu present.
***Metasoma***: terga I and II costate, median carina complete; tergite III costate on anterior 2/3, median carina incomplete; ovipositor short, about 0.25 × length of hind basitarsus.
  **♂.---Unknown**
  **Holotype**
    ♀: Ecuador, Napo Province, Isla de las Palmas, S 00°32.7′, W 077°52.7′, 1883 meters, October 12, 2006, reared from a mummy at Yanayacu Biological Station from an undetermined geometrid collected from an *Ocotea sp.* (Lauraceae), voucher *#* 18345. Deposited at the University of Wyoming Insect Museum (UWIM).
  **Distribution**
    Known only from the type locality.
  **Biology**
    This species has been reared from an undetermined geometrid mummy collected from an *Ocotea sp.* (Lauraceae). The specimen was collected as a mummy on the September 19, 2006 and emerged October 12, 2007.
  ***Mummy***: length = 11.0 mm; entire mummy mottled with gray, brown and black; thorax compact and wrinkled; glue hole located ventrally on the thorax; exit hole irregular, located postero-dorsally (Figure 4).
  **Comments**
    *Aldodes adydis* belongs in the *circumscriptus/gastritor* speciesgroup, differing from previously described species in those groups by having a predominately honey brown thorax and black abdomen, and the lack of yellow coloration on the second metasomal tergite. This species differs from *A. atnpileatus, A. albiterminus* and *A. arbitrium* in having the ocell-ocular distance less than width of the lateral ocellus and from *A. speciosus* by the absence of the median carina on the propodeum.
  **Etymology**
    The specific name is from the Latin *adydis,* meaning “small javelin or spear”, in reference to the short ovipositor of this species.

**Key to the Species of Aleiodes Reared from the Northeast Andes of Ecuador**
1. Metasomal tergite III entirely smooth and shiny (although scattered setae may be present); occipital carina entirely absent(Figures 13, 14) ***capillosus* n. sp.**

- Metasomal tergite III with various types of surface micro-sculpturing, never entirely smooth and shiny; occipital carina mostly present, although sometimes weak or absent at vertex 2
2(1). Central disc of mesopleuron entirely smooth and shiny; wings infuscate (Figures 25, 26) ***stilpnos* n. sp.**

- Central disc of mesopleuron with various types of surface micro-sculpturing, never entirely smooth and shiny; wings hyaline 3
3(2). Apex of hind tibia with a row of flattened setae along inner margin, forming a distinct flattened fringe 4
- Apex of hind tibia without a row of flattened setae along inner margin, setae when present never flattened or forming a distinct fringe 5
4(3). Occipital carina weak or absent at vertex; metasomal tergite 1 black to dark brown (Figures 16, 17) **greeneyi n. sp.**
- Occipital carina complete and well defined at vertex; antero-medial portion of metasomal tergite 1 with an irregular off-white marking (Figures 19, 20) **nebulosus n. sp.**

5(3). Ocellus small, ocell-ocular distance equal to or greater than width of lateral ocellus 6
- Ocellus large, ocell-ocular distance less than width of lateral ocellus 8
6(5). Malar space slightly longer than width of mandibular base; ovipositor length distinctly less than length of hind basitarsus; head, including gena, mostly black to dark brown (Figures 8, 9) ***arbitrlum* n. sp.**

**Figure 5.**
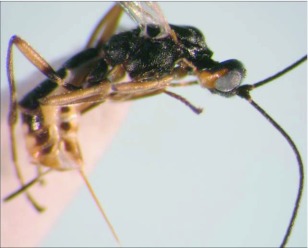
Lateral habitus of *A. albiterminus* n sp. Length 4.4 mm.

**Figure 6.**
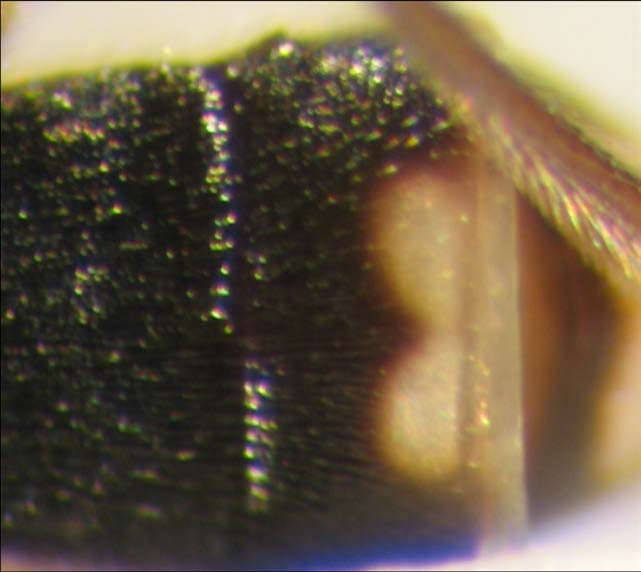
Second and third metasomal terga of *A. albiterminus* n sp. showing the half-heart shaped marking.

- Malar space at least 1.25 × width of mandibular base; ovipositor length variable; head color variable, gena light honey brown to whitish 7
7(6). Metasomal terga I and II with median carina present; ovipositor less than or equal to length of hind basitarsus; metasomal terga III and IV uniformly colored black, dark brown or light brown (Figure 11) ***atripileatus* n. sp.**

- Metasomal terga I and II with median carina absent; ovipositor roughly 2 × length of hind basitarsus; metasomal terga III and IV mostly black, postero-medial 1/2 of each respective tergite with an off-white mark in the shape of the top half of a heart (Figures 5, 6) ***albiterminus* n. sp.**

8(5). Malar space moderate, length 1.3 × width of mandibular base; median carina present on propodeum (Figures 22, 23). ***speciosus n. sp.***

- Malar space short, length 0.7 × width of mandibular base; median carina absent on propodeum (Figure 3) ***aclydis* n. sp.**

**Figure 7.**
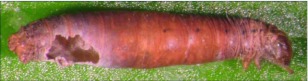
Host mummy of *A. albiterminus* n sp. Length, 6.8 mm.

***Aleiodes albiterminus* Townsend, new species (Figures 5–7)**
♀. ***Body color***: ground color of body black; gena, labial palpus and maxillary palpus honey brown; mandible mostly honey brown with a black tip; antennomere black; coxa, trochanter, trochantellus and femur of fore and mid leg dull yellow to light brown; tibia and tarsus of fore and mid leg brown; coxa and trochanter of hind leg black; basal ¾ of each trochantellus black, apical ¼ off-white; basal ⅓ of hind femur black to dark brown, apical ⅔ honey brown; tibia and tarsus of hind leg brown; mesosoma black; metasomal terga I and II black; terga III and IV mostly black, postero-medial ½ with 2 irregular off-white spots forming a ½ heart shape (Figure 6); remaining terga off-white medially, dark brown laterally; lateral terga off-white; ovipositor honey brown, basal ⅓ of ovipositor sheath white to off-white, apical ⅔ black (Figure 5).
***Body length***: = 4.4mm; fore wing length = 4.5 mm.
***Head***: antenna comprising 33 antennomeres, flagellomeres all longer than wide; malar space moderate, 1.25 × width of mandibular base and 0.4 × eye height; occipital carina weak or absent at vertex (Figure 17), well defined elsewhere, meeting hypostomal carina; oral space small and circular, diameter equal to malar space; clypeus swollen; ocellus small, ocell-ocular distance 1.3 × diameter of lateral ocellus; head granulate except occiput that is mostly smooth and shiny, dorso-mesad portion of occiput granulate; maxillary palpus not swollen; tips of mandibles overlapping when closed.
***Mesosoma***: pronotum foveate-foveolate; mesonotum and scutellum granulate, scutellar sulcus with a median carina, notauli weak and incomplete; mesopleuron granulate, subalar sulcus with irregular fovea, sternaulus absent; propodeum mostly granulate, dorso-posterior ⅕ sub-strigulate to irregular sculpturing, median carina present but incomplete.
*Leg:* tarsal claw simple.
***Wing***:(fore wing) vein r about ½ vein 3RSa; vein RS+Mb roughly equal to vein r; vein 1cu-a beyond vein IM by distance slightly more than 3.0 × vein 1 cu-a. (Hind wing) vein RS weakly sinuate, marginal cell narrowest in middle; vein r-m 0.70 × vein 1M; vein M+CU slightly longer than vein 1M; vein m-cu absent.
***Metasoma***: tergite I mostly granulate, costate on postero-medial ⅕, median carina absent; fused terga II and III granulate-costate, median carina absent; remainder of terga granulate; ovipositor long about 2.0 × length of hind basitarsus.
  **♂. Unknown**
  **Holotype**
    ♀: Ecuador, Napo Province, Rio Chalpi Grande, S 00° 21.6′, W 78°05.1′, 2837meters, December 16, 2005, reared from a geometrid feeding on *Alnus acuminatae* (Betulaceae), voucher # 10863. Deposited at the UWIM.
  **Distribution**
    Known only from the type locality.
  **Biology**
    This species was reared as a solitary parasitoid from an unknown geometrid feeding on *Alnus acuminata* (Betulaceae). The host caterpillar was collected on December 16, 2005, the wasp pupated on December 31, 2005 and emerged January of 2007.
  **Mummy**
    length = 6.8 mm; head capsule and abdomen light brown, thorax darker brown and wrinkled and compact; exit hole irregular, located postero-laterally (Figure 7). To the best of the author's knowledge, all recorded cases of *Aleiodes* mummies report the exit hole location being postero-dorsal. This may be unique to *A. albiterminus* or may be an artifact of being reared in an artificial environment. Additional rearings need to take place to elucidate this.
  **Comments**
    *Aleiodes albiterminus* belongs in the *circumscriptus/gastritor* species-group, differing from previously described species in those groups by the predominately black body color, and lack of yellow coloration medially on the second metasomal terga. This species differs from *A. speciosus* and *A. aclydis* in having the ocell-ocular distance greater than the width of the lateral ocellus and from *A. albiterminus* and *A. atripileatus* by the absence of the median carina on metasomal terga I and II.
  **Etymology**
    The specific name is from the Latin *albus* meaning “white” and the Latin *terminus,* meaning “end,” in reference to the white tip on the dorsal surface of the abdomen.

**Figure 8.**
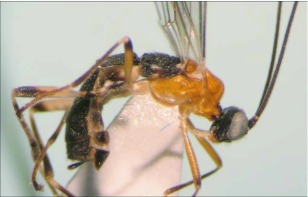
Lateral habitus of *A. arbitrium* n sp. Length, 5.7 mm.

**Figure 9.**
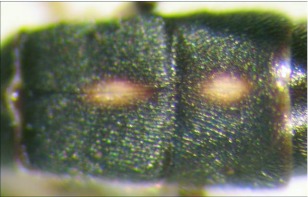
Second and third metasomal terga of *A. arbitrium* n sp.

**Figure 10.**
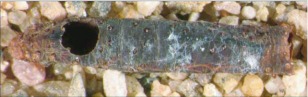
Host mummy of *A. arbitrium* n sp. Length, 10.2 mm.

***Aleiodes arbirium* Townsend, new species (Figures 8–10)**
♀. ***Body color***: head mostly black; 2 brown crescent moon-shaped marks on vertex bordering the eyes; oral space and posterior margin of gena brown; mandible black; maxillary palpus and labial palpus light brown; antenna black; coxa, trochanter and trochantellus of fore and mid leg light brown to off white; femur of fore and mid leg mostly light brown to off white, dark brown at apical tip; tibia of fore and mid leg mostly dark brown, off white at the basal tip; tarsus of fore and mid leg brown; hind coxa off white on basal ½, dark brown on apical ½; trochanter of hind leg mostly off white, dark brown at apical tip; trochantellus of hind leg dark brown; femur of hind leg off white on basal ⅔, dark brown on apical ⅓; first tarsomere of hind leg off white at basal tip, remainder brown to dark brown; remainder of tarsomeres of hind leg brown; pronotum dark brown medially, remainder honey brown; mesonotum and scutellum brownish orange; mesopleuron brownish orange on dorsal ⅔, off white on ventral ⅓; propodeum black; metasoma mostly black, terga II and III each with an off white, oval shaped marking medially (Figure 9); ovipositor sheath black, ovipositor light brown (Figure 8).
***Body length***: = 5.7 mm; fore wing length = 5.9 mm.
***Head***: antenna comprising 43 antennomeres, all flagellomeres greater than 2.0 × as long as wide; malar space slightly larger than width of mandibular base; occipital carina complete but weak at vertex, meeting hypostomal carina; oral space circular, slightly smaller than width of mandibular base; clypeus swollen; ocellus small, ocellocular distance slightly larger than diameter of lateral ocellus; head granulate; maxillary palpus not swollen; tips of mandibles overlapping when closed.
***Mesosoma***: pronotum mostly granulate with irregular fovea postero-laterally; mesonotum mostly granulate with irregular costae postero-medially, notauli present but incomplete; scutellum granulate; mesopleuron granulate, epicnemial carina present, sternaulus absent; propodeum granulate on apical ½, rugulose to rugose on basal ½, median carina present.
***Leg***: tarsal claw simple.
***Wing***: (fore wing) vein r about 0.4 × vein 3RSa; vein RS+Mb about 0.6 × vein r; vein cu-a beyond vein 1M by about 3.0 × vein cu-a. (Hind wing) vein RS slightly sinuate, thus marginal cell narrowest in the middle; vein r-m about 0.5 × vein 1M; vein M+CU longer than vein 1M; vein m-cu present.
***Metasoma***: terga I-III costate, median carina complete to the end of tergite II (Figure 9); ovipositor about 0.7 × length of hind basitarsus.
  **♂. --- Essentially as in ♀ except antenna with 41 antennomeres.**
  **Holotype**
    ♀: Ecudar, Napo Province, Yanayacu Biological Station S 00°35.9′ W 77°53.4′, 2163 meters, July 10,2006, reared from an unknown geometrid collected from *Diplazium costale* (Dryopteridaceae), voucher # 14608. Deposited at the UWIM.
  **Paratypes**
    Ecuador, Napo Province, Yanayacu Biological Station S 00°35.9′ W 77°53.4′, 2163 meters; 1 ♂, October 17, 2007, reared from a *Hypena* sp. (Noctuidae) on *Phenax rugosus* (Urticaceae), voucher # 29168; 1 ♂, August 21, 2007, reared from a geometrid on *Diplazium costale* (Dryopteridaceae), voucher # 25393. Deposited at the UWIM.
  **Distribution**
    Known only from the type locality.
  **Biology**
    Two specimens *of Aleiodes arbitnum* were reared from 2 individuals of an undetermined geometrid feeding on *Diplazium costale* (Dryopteridaceae) and one specimen of *A. arbitnum* was reared from a *Hypena* sp. (Noctuidae) feeding on *Phenax rugosus* (Urticaceae).
  ***Mummy***: length = 10.2 mm; decapitated, entirely dark brown; exit hole located postero-dorsally (Figure 10).
  **Comments**
    *Aleiodes arbitrium* belongs in the *circumscriptus/gastritor* species-group, differing from previously described species in those groups by the contrasting coloring of the predominately honey brown thorax and black abdomen, and by lack of yellow coloration medially on the second metasomal tergite. This species differs from *A. speciosus* and *A. aclydis* in having the ocell-ocular distance greater than the width of the lateral ocellus, and from *A. albiterminus* and *A. atripileatus* in the head being almost entirely brown, in having a short malar space, and in that the ovipositor is significantly shorter than the length of the hind basitarsus.
  **Etymology**
    The specific name is from the Latin *arbitnum,* meaning “mastery or power,” in reference to the ability of the species to master developing within a living host and being powerful enough to overcome host immune responses.

**Figure 11.**
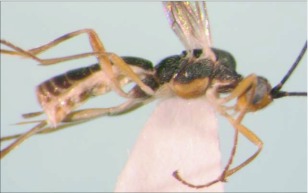
Lateral habitus of *A. atripileatus* n sp. Length, 4.2 mm.

**Figure 12.**
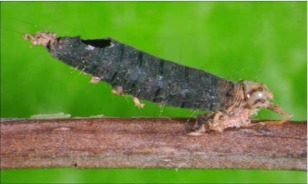
Host mummy of *A. atripileatus* n sp. Length, 7.7 mm.

***Aleiodes atripileatus* Townsend, new species (Figures 11, 12)**
♀. ***Body color:*** ground color of head honey brown; vertex and ocellar triangle black; occiput with large black semi-circular spot behind vertex; scape dark brown pedicel dark brown with light brown at the apex; flagellomere dark brown to black; tip of mandible dark brown; pronotum, mesonotum, scutellum and propodeum black to dark brown; dorsal half of mesopleuron black, ventral ½ honey brown; leg mostly honey brown; coxa and trochanter of fore and mid leg white to light brown; coxa of hind leg mostly dark brown with a dorsal longitudinal white stripe, apical ¼ of coxa white to light brown; metasomal terga I-IV black to dark brown; ovipositor sheath dark brown, ovipositor brown (Figure 11).
***Body length***: = 4.2 mm; fore wing length = 4.0 mm.
***Head***: antenna comprising 38 antennomeres, flagellomeres roughly 2.0 × as long as wide; malar space moderate, length 1.5 × basal width of mandible, and approximately ½ eye height; occipital carina weak or absent at vertex, well defined elsewhere, reaching hypostomal carina; oral space small and circular, maximum width slightly smaller than basal width of mandible; clypeus not swollen; ocellus small, ocell-ocular distance 1.7 × diameter of lateral ocellus; head granulate; maxillary palpus not swollen; tips of mandibles touching when closed.
***Mesosoma***: pronotum mesonotum and scutellum granulate; notauli very weak; mesopleuron granulate, sternaulus present; propodeum rugulose-granulate, median carina complete, areola absent.
***Leg***: tarsal claw not pectinate.
***Wing***: (fore wing) vein r approximately ⅓ vein 3RSa, second submarginal cell nearly rectangular; vein 1cu-a beyond vein 1M by distance slightly less than 2.0 × vein 1cu-a; vein RS+Mb about the same size as vein r. (Hind wing) vein RS slightly sinuate, marginal cell narrowest in middle; vein r-m shorter than vein 1M; vein M+CU about equal to 1M; vein m-cu absent.
***Metasoma***: tergite I granulate-costulate anteriorly, costulate posteriorly, median carina complete; tergite II costulate, median carina complete; tergite III granulate-costulate anteriorly, granulate posteriorly, median carina incomplete; ovipositor slightly longer than the length of hind basitarsus.
  **♂. Essentially as in ♀ but antenna with 36 antennomeres instead of 38 or 39.**
  **Variation**
    Body length = 4.1 mm to 4.8 mm; fore wing length = 3.9 mm to 4.5 mm; antennomeres 36 to 39; ocell-ocular distance 1.5 to 1.7 × width of lateral ocellus; malar space 1.3 to 1.5 × width of mandibular base; ovipositor length slightly shorter than hind basitarsus length to slightly longer than hind basitarsus length; sculpturing on terga I-III varies from being almost entirely granulate to being almost entirely costulate.
  **Holotype**
    ♀: Ecuador, Napo Province, Yanayacu Biological Station, S 00°35.9′, W 77°53.4′, 2163 meters, June 20, 2006, reared from a *Hypena sp.* (noctuid) feeding on *Boehmeria caudata* (Urticaceae), voucher # 15235. Deposited at the UWIM.
  **Paratypes**
    Ecuador, Napo Province, Yanayacu Biological Station S 00°35.9′ W 77°53.4′, 2163 meters; 1 ♂, Jan 31, 2005, reared from a noctuid feeding on a *Miriocarpa* sp. (Urticaceae), voucher # 1608; 1 ♂*,* January 24, 2005, reared from a noctuid feeding on an Urticaceae sp., voucher # 1504; 1 ♂*,* September 12, 2007, reared from a noctuid feeding on a *Phenax* sp. (Urticaceae), voucher # 26164; 1 ♀, August 22, 2005, reared from a noctuid feeding on *Phenax rugosus* (Urticaceae), voucher # 6232; 1 ♀, June 02, 2006, reared from a noctuid feeding on *Phenax rugosus* (Urticaceae), voucher # 15131; 1 ♀, August 25, 2005, reared from a noctuid feeding on an Urticaceae sp., voucher # 6474; 1 ♀, January 31, 2005, reared from a noctuid feeding on *Phenax rugosus* (Urticaceae), voucher # 1612; 1 ♀, May 22, 2005, reared from a collected noctuid mummy on an Urticaceae sp., voucher # 2806; 1 ♀, January 11, 2007, reared from a collected noctuid mummy, voucher # 19949; 1 ♀, November 12, 2007, reared from a noctuid feeding on *B. bullata* (Urticaceae), voucher # 28333. All specimens deposited at the UWIM.
  **Distribution**
    Known only from the type locality.
  **Biology**
    All *Aleiodes atripileatus* specimens have been reared from noctuid hosts on Urticaceae host plants. One specimen was reared from an unidentified *Hypena* species (noctuid) feeding on a *Miriocarpa* species (Urticaceae). The remaining specimens of *A. atripileatus* were reared from what appears to be the same *Hypena sp.* host based on the morphology of the mummies; however the caterpillars were not identified past family level when alive so other genera of noctuids cannot be definitively ruled out. In addition to the *Minocarpa sp.* plant host, *A. atripileatus* was reared from hosts feeding on *Phenax rugosus* (Urticaceae), *B. bullata* (Urticaceae) and three other unidentified species of Urticaceae.
  ***Mummy***: length = 7.3 to 8.3 mm; head capsule either entirely light brown, or light brown with irregular dark brown markings, thorax wrinkled, compact and light to dark brown, abdominal prolegs light brown, abdomen with many small, light brown, circular protuberances, remainder of abdomen black; glue hole located on the venter of the thorax; mummy glued to substrate forming approximately a 10 to 15 degree angle; exit hole circular, located postero-dorsally (Figure 12).
  **Comments**
    *Aleiodes atripileatus* belongs in the *circumscriptus/gastritor* species-group, differing from previously described species in those groups by having a multi-colored pattern and lacking yellow coloration medially on the second metasomal tergite. This species differs from *A. speciosus* and *A. aclydis* by having the ocell-ocular distance greater than the width of the lateral ocellus and from *A. arbitnum* and *A. albiterminus* by having the third and fourth metasomal terga uniformly colored black or dark brown.
  **Etymology**
    The specific name is from the Latin *atrum,* meaning “black,” and the Latin *pileatus,* meaning “capped,” in reference to the large black spot on the vertex and occiput of the head.

**Figure 13.**
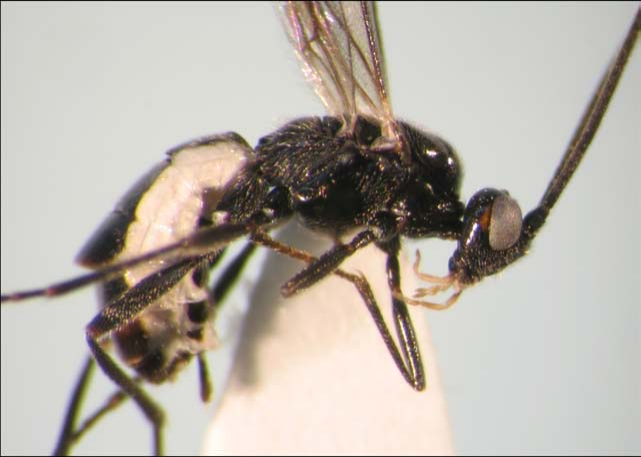
Lateral habitus of *A. capillosus* n sp. Length, 5.6 mm.

**Figure 14.**
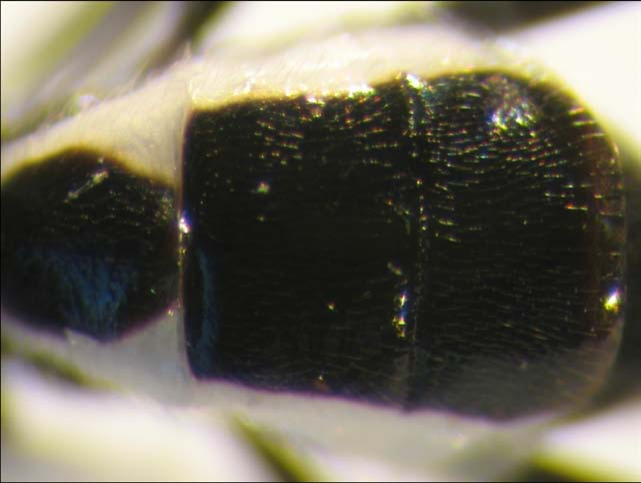
First through third metasomal terga of *A. capillosus* n sp.

**Figure 15.**
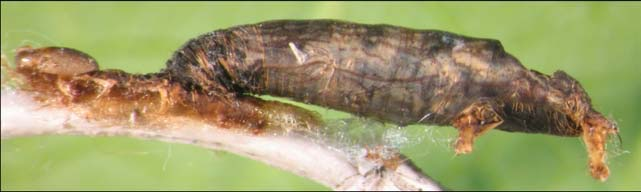
Host mummy of *A. capillosus* n sp. Length, 11.0 mm.

***Alelodes capillosus* Townsend, new species (Figures 13–15)**
♀. ***Body color***: body mostly black; labial palpus light brown; maxillary palpus dark brown basally, light brown apically; dark brown irregular spot on gena on posterior margin of eye; antenna black; metasomal tergite I with a large black oval spot medially spanning from the anterior to posterior edges of the tergite, remainder of tergite I white; terga II and III black medially, white laterally (Figure 14); remainder of terga dark brown to black medially, white laterally; lateral terga white, abdominal sterna white with 4 paired black spots; wings lightly infuscate (Figure 13).
***Body length***: = *5.6* mm; fore wing length = 5.3 mm.
***Head***: antenna comprising 42 antennomeres, flagellomeres longer than wide; malar space moderate, length 1.8 × basal width of mandible and ½ eye height; occipital carina absent; oral space circular, 1.3 × width of mandibular base; clypeus not swollen; ocellus small, ocell-ocular distance 2.0 × size of lateral ocellus; head mostly granulate, face with rugae below antenna; maxillary palpus not swollen; mandible small.
***Mesosoma***: pronotum granulate with a few rugae medially; mesonotum and scutellum granulate, notauli weak, present on apical ⅓ only; mesopleuron finely granulate antero-dorsally, remainder smooth, epicnemial carina absent; propodeum finely granulate, appearing mostly smooth; propodeum and metapleuron with long white setae.
*Leg:* hind coxa with long white setae, tarsal claw not pectinate.
***Wing***: (fore wing) vein r about ½ vein 3RSa; vein lcua beyond vein 1M by distance 1.5 × vein 1cu-a; vein 1Cub 1.5 × vein 1CUa. (Hind wing) vein RS slightly sinuate, marginal cell narrowest in the middle; vein M+CU slightly longer than vein 1M; vein m-cu absent.
***Metasoma***: terga I-IV smooth and shiny (Figure 14), although scattered setae are present, median carina absent; remainder of terga finely granulate; ovipositor slightly longer than hind basitarsus.
  **♂. Essentially as in ♀ except body length 5.0 mm, fore wing length 4.6 mm, 45 antennomeres and wings heavily infuscate.**
  **Holotype**
    ♀: Ecuador, Napo Province, Papallacta, S 00° 21.8′, W 78° 05.1′, 2800 meters, July 20, 2006, reared at Yanayacu Biological Station from an unknown geometrid, voucher # 16343. Deposited at the (UWIM).
  **Paratype**
    ♂: Ecuador, Napo Province, Yanayacu Biological Station, S 00°35.9′, W 77°53.4′, 2163 meters, June 7, 2006, reared from an unknown geometrid mummy collected on *Diplazium vesiculosum* (Dryopteridaceae), voucher # 14723. Deposited at the UWIM.
  **Distribution**
    Known only from the type locality.
  **Biology**
    Reared from mummies of two unknown species of geometrid collected from *D. vesiculosum* (Dryopteridaceae), and an unknown plant respectively. The 2 specimens emerged on July 20, 2006 and June 7, 2006 respectively.
  ***Mummy***: length = 8.0 to 11.0 mm; head capsule light brown to dark brown, remainder of mummy mottled light brown, brown, and black; exit hole located posterodorsally, in one specimen glued flat to substrate under the thorax producing essentially no angle (Figure 15).
  **Comments**
    This species clearly belongs in the *gressitti* species-group as it has the defining features of the group: metasomal terga and propodeum very finely coriaceous and shining, appearing smooth, fore wing vein 1cu-a beyond 1M by 1.5 × length of 1cu-a, and hind wing vein RS slightly sinuate. *Aleiodes capillosus* differs from the only other described species in the *gressitti* species-group in the new world, *A. lissos,* by having a stark black and white color pattern, and by lacking the occipital carina. This is the first recorded species in the *gressitti* species-group from the Neotropics.
  **Etymology**
    The specific name is from the Latin *capillosus,* meaning “hairy,” referernce to the conspicuous white setae present on this species, especially on the hind coxa, metapleuron and propodeum.

**Figure 16.**
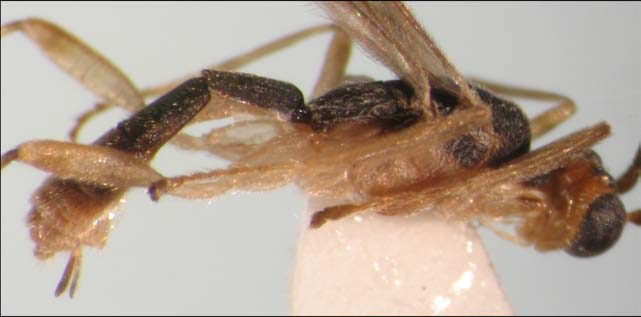
Lateral habitus of *A. greeney* n sp. Length, 4.9 mm.

**Figure 17.**
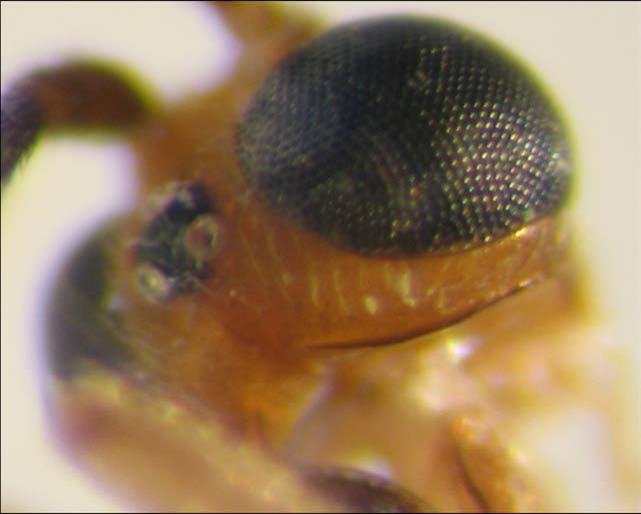
Postero-dorsal view of the head of *A. greeneyi* n sp. showing the absence of the occipital carina at the vertex.

**Figure 18.**
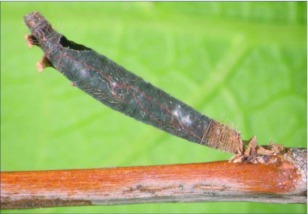
Host mummy of *A.* *greeneyi* n sp. Length, 9.9 mm.

***Aleiodes greeneyl* Townsend, new species (Figures 16–18)**
♀. ***Body color***: head mostly brown, ocellar triangle black; maxillary palpus and labial palpus light brown; antenna dark brown; leg mostly light brown; fifth tarsomere on each leg dark brown; pronotum black; mesonotum mostly black with a light brown square postero-mesad; scutellum dark brown to black laterally, light brown medially; scutellar sulcus dark brown; propodeum black; mesopleuron and metapleuron light brown; metasomal terga I and II black; terga III and IV dark brown laterally, light brown medially; remainder of terga light brown; ovipositor sheath dark brown (Figure 16).
***Body length***: = 4.9 mm; fore wing length = 4.2 mm.
***Head***: antenna comprising 42 antennomeres, all twice as long as wide; malar space moderate, 1.25 × width of mandibular base; occipital carina weak or absent at vertex, well defined elsewhere (Figure 17), meeting hypostomal carina; oral space small, diameter 0.8 × width of mandibular base; clypeus normal, not swollen; ocell-ocular distance roughly equal to diameter of lateral ocellus; head granulate; maxillary palpus not swollen; tips of mandibles overlapping when closed.
***Mesosoma***: pronotum mostly granulate with irregular fovea; mesonotum and scutellum granulate, notauli present but incomplete; mesopleuron granulate, sternaulus absent, epicnemial carina present; propodeum mostly punctate with irregular fovea posteriorly, median carina incomplete.
*Leg:* apex of hind tibia with a row of flattened setae along inner margin; tarsal claw simple.
***Wing***:(fore wing) with vein r 0.5 × vein 3RSa; vein RS+Mb slightly less than vein r; vein 1cu-a beyond vein 1M by 3.0 × vein 1cu-a. (Hind wing) vein RS slightly sinuate, marginal cell narrowest in the middle; vein r-m slightly less than length of vein 1M; vein M+CU 1.33 × vein 1M; vein m-cu present.
***Metasoma***: terga I and II, median carina complete; tergite III mostly costulate, postero-medial portion granulate, median carina incomplete; ovipositor short, about 0.5 × length of hind basitarsus. ♂. Unknown.
**Holotype**
  ♀: Ecuador, Napo Province, Isla de Las Palmas, S 00°32.7′, W 077°52.5′, 1885 m, Dec 23, 2005, reared at Yanayacu Biological Station from an unidentified geometrid feeding on *Evodianthus funifer* (Cyclanthaceae), voucher #9714. Deposited at the UWIM.
**Distribution**
  Known only from the type locality.
**Biology**
  *Aleiodes greeneyi* has been reared from an unidentified species of geometrid feeding on *Evodianthus funifer* (Cyclanthaceae). The host caterpillar was collected on November 19, 2005, and the wasp on December 2, 2005 and emerged December 23, 2005.
***Mummy***: length = 9.9 mm; head capsule and prolegs light brown, thorax with light brown “wrinkles” over black base, remainder of mummy black, thorax wrinkled and compact; glue hole located ventrally on first thoracic segment, glued mummy at approximately a 45 degree angle from the substrate; exit hole located posterodorsally (Figure 18).
**Comments**
  This species belongs in the *senatus* species-group based on the presence of a row of flattened setae along the inner margin of the apex of the hind tibia. It differs from previously described Neotropical species in this species-group, *Aleiodes nigrestemmaticum* (Enderlein), *A. nigribasis* (Enderlein) and *A. bakeri* (Brues), by being predominantly black colored. This species differs from *A. nebulosus* in having the occipital carina weak or absent at the vertex and having a unicolored metasomal tergite I.
**Etymology**
  This species is named after Harold F. Greeney, founder and owner of Yanayacu Biological Station, to honor his relentless pursuit to better understand and document the natural history of Neotropical organisms.

**Figure 19.**
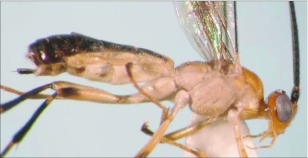
Lateral habitus of *A.* *nebulosus* n sp. Length, 6.1 mm.

**Figure 20.**
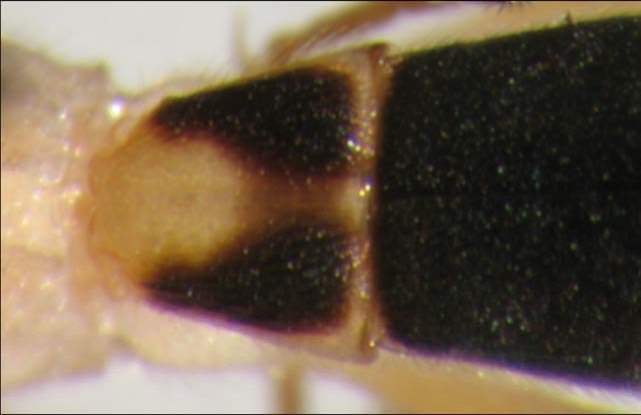
Posterior portion of propodeum and first and second metasomal terga of *A. nebulosus* n sp.

**Figure 21.**
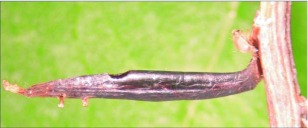
Host mummy of *A.* *nebulosus* n sp. Length, 18.0 mm.

***Aleiodes nebulosus* Townsend, new species (Figures 19–21)**
♀. ***Body color***: head mostly light brown, ocellar triangle black; mandible mostly off-white, tips dark brown; maxillary palpus and labial palpus off-white to light brown; antenna black; coxa and trochanter white to off-white; trochantellus off-white to light brown; femur mostly light brown with a dark brown spot apically on the outer margin; fore and mid tibia light brown, hind tibia dark brown to black; first through fourth tarsomeres on fore and mid leg light brown, fifth tarsomere dark brown, tarsomeres of hind leg dark brown to black; pronotum, mesonotum and scutellum light brown; metanotum and propodeum cream to light brown; propleuron, mesopleuron and metapleuron off-white; metasomal tergite I mostly dark brown to black with an irregular off-white marking antero-medially; terga II and III black (Figure 20); remainder of terga dark brown; lateral terga off- white to light brown; abdominal sterna mostly off white to light brown, last 4 sterna with paired brown spots; ovipositor light brown, ovipositor sheath off-white at the basal tip, remainder black (Figure 19).
***Body length***: = 6.1 mm; fore wing length = 5.5 mm.
***Head***: antenna comprising 47 antennomeres, all flagellomeres roughly equal to or slightly longer than 2.0 × width; malar space moderate, length about 1.2 × width of mandibular base; occipital carina well defined and complete, meeting hypostomal carina; oral space small and circular, diameter slightly smaller than width of mandibular base; clypeus swollen; ocellus moderate, ocell-ocular distance 1.25 × diameter of lateral ocellus; head granulate; maxillary palpus not swollen; tips of mandibles overlapping when closed.
***Mesosoma***: pronotum mostly granulate, with irregular fovea laterally; mesonotum and scutellum granulate, notauli present, but incomplete; mesopleuron granulate, epicnemial carina present, sternaulus present but weak; propodeum granulate anteriorly, rugulose posteriorly, median carina complete.
***Leg***: apex of hind tibia with a row of flattened setae along inner margin; tarsal claw simple.
***Wing***:(fore wing) vein r about 0.8 × vein 3RSa; vein RS+Mb about 0.7 × vein r; vein 1cu-a beyond vein 1M by distance slightly less than 2.5 × vein 1cu-a. (Hind wing) vein RS parallel to wing margin along basal ½, gradually curved downward apically so marginal cell widened towards the apex; vein r-m about 0.8 × vein 1M; vein M+CU shorter than vein 1M; vein m-cu present.
***Metasoma***: terga I and II costate, median carina complete to end of tergite II; tergite III costate on anterior ⅔, median carina absent; ovipositor short, length about 0.33 × length of the hind basitarsus.
  **♂. Unknown**
  **Holotype**
    ♀: Ecuador, Napo Province, Isla de Las Palmas, S 00°32.7′, W 077°52.5′, 1885 m, October 12, 2006, reared from a noctuid feeding on *Acalypha platyphylla* (Euphorbiaceae), voucher # 17714. Deposited at the UWIM.
  **Distribution**
    Known only from the type locality.
  **Biology**
    *Aleiodes nebulosus* has been reared from an undetermined noctuid feeding on *Acalypha platyphylla* (Euphorbiaceae). The host caterpillar was collected on August 15, 2006 and the wasp emerged on October 12, 2007. One female *Mesochorus sp.* (Ichneumonidae: Mesochorinae) hyperparasitoid was reared from a mummy with the same characteristics as that of *A. nebulosus.*
  ***Mummy***: length =18 mm; head capsule and prolegs light brown, remainder black, thorax wrinkled and compact; glue hole located on the venter of the thorax, mummy glued to substrate forming a 90 degree angle; exit hole located posterior to approximate midpoint of abdomen (Figure 21).
  **Comments**
    This species belongs in the *seriatus* species-group based on the presence of a row of flattened setae along the inner margin of the apex of the hind tibia. It differs from previously described Neotropical species in this species-group, *Aleiodes nigrestemmaticum* (Enderlein), *A. nigribasis* (Enderlein) and *A. bakeri* (Brues), in having the metasomal terga II-VIII black to dark brown. This species differs from *A. greeneyi* by having the occipital carina complete and by having a bi-colored metasomal tergite I.
  **Etymology**
    The specific name is from the Latin *nebulosus,* meaning “cloudy,” in reference to the cloud forest habitat that this species lives in.

**Figure 22.**
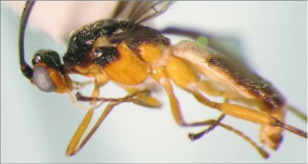
Lateral habitus of *A.* *spedosus* n sp. Length, 4.9 mm.

**Figure 23.**
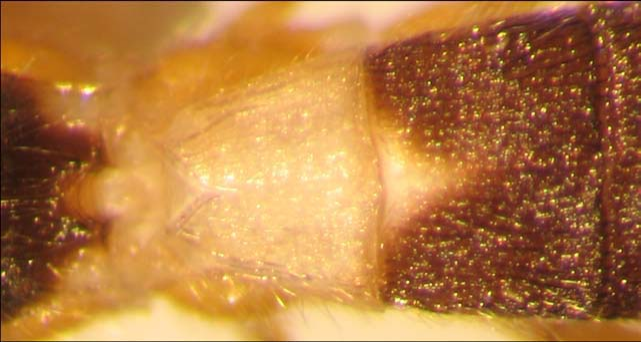
Posterior portion of propodeum and first and second metasomal terga of *A. speciosus* n sp.

**Figure 24.**
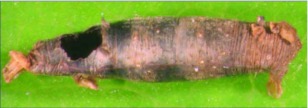
Host mummy of *A. speciosus* n sp. Length, 7.8 mm.

***Aleiodes speciosus* Townsend, new species (Figures 22–24)**
♂. ***Body color***: ground color of head honey brown; dorsal ½ of occiput, vertex, ocellar triangle, frons, and medial portion of face black; maxillary palpus and labial palpus honey brown; mandible mostly honey brown, tip dark brown; antenna silver-black; leg mostly honey brown; tibia and tarsus of fore and mid leg light brown, tibia and tarsus of hind leg dark brown; mesonotum, scutellum, and metanotum black; propodeum dark brown; mesopleuron mostly honey brown with a large black mark on antero-dorsal ¼; metasomal tergite I white; tergite II mostly brown with a white spot antero-mesad in the shape of an isosceles triangle (Figure 23); terga III and IV brown, terga V and VI off-white; remainder of abdomen brown (Figure 22).
***Body length***: = 4.9 mm; fore wing length = 4.5 mm.
***Head***: antenna comprising 42 antennomeres, all flagellomeres roughly equal to or slightly longer than 2.0 × their width; malar space moderate, length about 1.3 × width of mandibular base; occipital carina well defined except at vertex, meeting hypostomal carina; oral space small and circular, diameter roughly equal to width of mandibular base; clypeus swollen; ocellus large, ocellocular distance 0.6 × diameter of lateral ocellus; head granulate; maxillary palpus not swollen; tips of mandibles overlapping when closed.
***Mesosoma***: pronotum mostly granulate with irregular fovea laterally; mesonotum and scutellum granulate, notauli present but incomplete; mesopleuron mostly smooth, rugose on dorsal ¼; propodeum granulate anteriorly, rugose posteriorly, median carina complete.
***Leg***: tarsal claw simple.
***Wing***: (fore wing) with vein r about 0.5 × vein 3RSa; vein RS+Mb about 0.7 × vein r; vein 1cu-a beyond vein 1M by distance 3.0 × vein 1cu-a. (Hind wing) vein RS slightly sinuate, marginal cell narrowest in the middle; vein r-m about 0.5 × vein 1M; vein M+CU longer than vein 1M; vein m-cu present.
***Metasoma***: terga I and II costate, median carina complete; tergite III costate on anterior ⅔, median carina present but incomplete.
  **♀. --- unknown**
  **Holotype**
    ♂: Ecuador, Napo Province, Camino a Loreto, S 00°43.2′, W 77°45.5′, 1383m, August 27, 2005, collected as a mummy (undetermined Lepidoptera) on a *Miconia sp.* (Melostomataceae), voucher # 5853. Deposited at the UWIM.
  **Distribution**
    Known only from the type locality.
  **Biology**
    This species has been collected and reared from an unidentifiable Lepidoptera larva feeding on a *Miconia sp.* (Melostomataceae). The host caterpillar was collected on August 4, 2005 and the wasp emerged on August 27, 2005.
  ***Mummy***: length = 7.8 mm; decapitated, mostly dark brown to black, prolegs brown; exit hole located posterodorsally (Figure 24).
  **Comments**
    *Aleiodes speciosus* belongs in the *circumscriptus/gastritor* species-group, differing from previously described species in those groups by having a predominately black body with a white metasomal tergite I and lacking yellow coloring on metasomal tergite II. This species differs from *A. atripileatus, A. albiteminus* and *A. arbitrium* in having the ocell-ocular distance less than the width of lateral ocellus and from *A. aclydis* by having a median carina on the propodeum, and having a white metasomal tergite I.
  **Etymology**
    The specific name is from the Latin *speciosus,* meaning “beautiful or splendid.”

**Figure 25.**
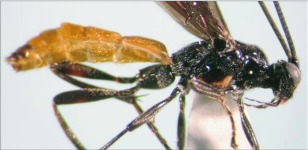
Lateral habitus of *A. stilpnos* n sp. Length, 9.0 mm.

**Figure 26.**
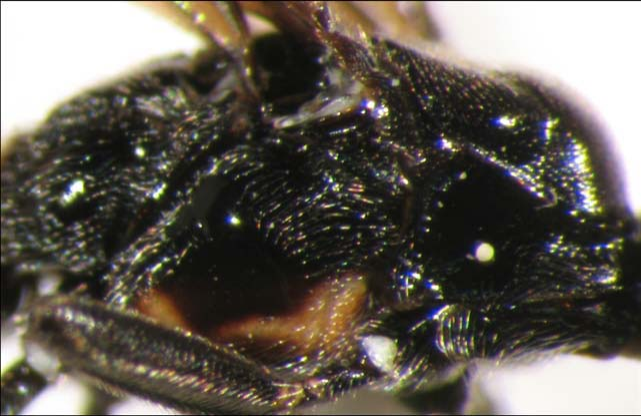
Lateral view of the thorax of *A. stilpnos* n sp. showing the smooth central disc of the mesopleuron.

**Figure 27.**
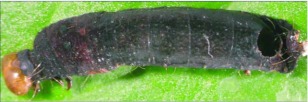
Host mummy of *A.* *stilpnos* n sp. Length, 17.0 mm.

***Aleiodes stilpnos* Townsend, new species (Figures 25–27)**
♂. ***Body color***: head black; labial palpus and maxillary palpus brown; antenna silver-black; mesosoma mostly black; dorsal ½ of mesopleuron black, ventral ½ with a dark brown mark medially on the upper ½, light brown elsewhere; leg mostly black, apical tip of trochanter and basal tip of femur brown; femur of hind leg with a brown spot medially; metasoma mostly dull orange, tip of abdomen dark brown; wings infuscate (Figure 25).
***Body length***: = 9.0 mm; fore wing length = 8.5 mm.
***Head***: antenna comprising 65 antennomeres, flagellomeres compact, all about 1 × as long as wide; malar space moderate, length about 1.3 × width of mandibular base; occipital carina well defined, meeting hypostomal carina; oral space small and circular, diameter slightly less than width of mandibular base; clypeus slightly swollen; ocellus moderate, ocell-ocular distance slightly longer than the diameter or lateral ocellus; face and frons costate-rugose, rest of head lightly granulate; maxillary palpus not swollen; tips of mandibles overlapping when closed.
***Mesosoma***: pronotum foveolate; mesonotum and scutellum mostly smooth and shining, notauli present and well defined to the end of mesonotum but not meeting, scutellar sulcus with multiple longitudinal carinae; central disc of mesopleuron smooth and highly polished (Figure 26), remainder setose, epicnemial carina present, sternaulus absent; propodeum rugose, median carina complete.
***Leg***: tarsal claw strongly pectinate; hind coxa swollen, balloon-like.
***Wing***: (fore wing) vein r about 0.5 × vein 3RSa; vein RS+Mb 0.7 × vein r; vein 1cu-a beyond vein M by about 2.0 × vein 1cu-a. (Hind wing) vein RS parallel to wing margin along basal ½, gradually curved downward so marginal cell is widened towards the apex; vein r-m 0.6 × vein 1M; vein M+CU shorter than vein 1M; vein m-cu absent.
***Metasoma***: terga I-III costate, median carina complete to the end of the tergite II.
  **♀. Unknown.**
  **Holotype**
    ♂: ECUADOR, Napo Province, Yanayacu Biological Station, S 00°35.9′, W 77°53.4′, 2163 meters, October 21, 2006, reared from an unknown noctuid feeding on *Polygonum punctatum* (Polygonaceae), voucher # 17925. Deposited at the UWIM.
  **Distribution**
    Known only from the type locality.
  **Biology**
    This species has been reared from an undetermined noctuid feeding on *Polygonum punctatum* (Polygonaceae). The host caterpillar was collected on August 25, 2006, the wasp pupated on September 8, 2006 and emerged on October 21,2007.
  ***Mummy***: length =17 mm; head capsule light brown, remainder dark brown to black; antero-ventral portion of abdomen slightly expanded, not decapitated; exit hole located postero-dorsally (Figure 27).
  **Comments**
    This species and *Aleiodes fuscipennis* (Szepligeti) are the only described species from the Neotropics in the *albitibia* species-group, defined as mesopleuron having a smooth and highly polished central disc. This species differs from *A. fuscipennis* by having a black mesosoma, having costate sculpturing on the first through third metasomal terga, having a complete median carina on the second metasomal tergite, and the ocell-ocular distance being longer than the width of the lateral ocellus. This species clearly belongs to the *albitibia* species-group, but the expansion in the antero-ventral portion of the host mummy is not as extreme as in other reared records. The mummy does have some expansion, however.
  **Etymology**
    The specific name is from the Greek *stilpnos,* meaning “glittering or shining,” in reference to the overall smooth and shining appearance of this species.

## Discussion

The species described in this study belong to four (*albitibia, circumscriptus/gastritor, gressitti* and *seriatus* groups) of the Aleiodes species-groups known to occur in the Neotropics: *albitibia, apicalis, circumscriptus/gastritor, coxalis, melanopterus, pallidator, praetor, pulchripes* and *seriatus* groups. *Aleiodes capillosus* represents the first distribution record of the *gressitti* species-group from the Neotropics. Speciesgroups known to occur in the Neotropics but not represented in this study may be absent for the following reasons: First, most *Aleiodes* are solitary and live at low population density levels. Second, the diversity of potential hosts is extremely high in the eastern Andes of Ecuador. Rearing from additional lepidopteran groups or increasing the number of caterpillar individuals reared within those groups, both of which might raise the probability of rearing *Aleiodes* from a greater diversity of species-groups, was beyond the scope of this study. Third, many host species, and thus the *Aleiodes* that parasitize them, may complete their entire life cycles in the canopy, which was not sampled in this study. In the future, it may be possible to sample fresh tree falls, or build platforms in the canopy. Although 28,310 caterpillars have been collected and reared at Yanayacu to date, lepidopteran and associated parasitoid diversity, including *Aleiodes,* has not been adequately sampled. However, the specimens sampled in this study significantly adds to the biological knowledge of Neotropical *Aleiodes,* as host documentation is provided for the 9 species herein described.

### Editor's note

Paper copies of this article will be deposited in the following libraries. Senckenberg Library, Frankfurt Germany; National Museum of Natural History, Paris, France; Field Museum of Natural History, Chicago, Illinois USA; the University of Wisconsin, Madison, USA; the University of Arizona, Tucson, Arizona USA; Smithsonian Institution Libraries, Washington D.C. USA; The Linnean Society, London, England.
